# Publisher Correction: Structural characterization of ice XIX as the second polymorph related to ice VI

**DOI:** 10.1038/s41467-021-23514-0

**Published:** 2021-05-14

**Authors:** Tobias M. Gasser, Alexander V. Thoeny, A. Dominic Fortes, Thomas Loerting

**Affiliations:** 1 Institute of Physical Chemistry, University of Innsbruck, Innsbruck, Austria; 2 ISIS Neutron and Muon Facility, Rutherford Appleton Laboratory, Harwell Science and Innovation Campus, Chilton, Oxfordshire OX11 0QX UK

**Keywords:** Chemical physics, Phase transitions and critical phenomena, Structure of solids and liquids

Correction to: *Nature Communications* 10.1038/s41467-021-21161-z, published online 18 February 2021.

The original version of this Article contained an error in the 14th sentence of the third paragraph of the ‘Refinement of ice XIX from neutron powder data’ section, which incorrectly read ‘once we became aware of the structure fitting reported by Yamane et al. on Research Square^36^’. The correct version states ‘once we became aware of the structure fitting reported by Yamane et al.^36^, first deposited as a preprint’ in place of the above.

The original version of this Article also contained an error in ref. 36, which incorrectly cited a preprint of the work: ‘Yamane, R. et al. Experimental evidence for the existence of a second partially-ordered phase of ice VI. Nat. Commun. https://www.researchsquare.com/article/rs-64673/v1 (2020)’. The correct form of ref. 36 is ‘Yamane, R. et al. Experimental evidence for the existence of a second partially-ordered phase of ice VI. *Nat. Commun*. **12**, 1129 (2021)’.

This has been corrected in the PDF and HTML versions of the Article.

