# Supplementary material for: Structural characterization of ice XIX as the second polymorph related to ice VI
Source: Nat Commun. 2021 Feb 18;12:1128. doi: 10.1038/s41467-021-21161-z (PMC7892819; doi:10.1038/s41467-021-21161-z)
Supplement: Supplementary file 1 — Supplementary Information [file 41467_2021_21161_MOESM1_ESM.pdf]

SUPPLEMENTARY INFORMATION

Structural characterization of ice XIX as the second polymorph related to ice VI

Tobias M. Gasser[1], Alexander V. Thoeny[1], A. Dominic Fortes[2], Thomas Loerting[1,*]

[1]*Institute of Physical Chemistry, University of Innsbruck, 6020 Innsbruck, Austria*

[2]*ISIS Neutron and Muon Facility, Rutherford Appleton Laboratory, Harwell Science and Innovation Campus, Chilton, Oxfordshire, OX11 0QX, UK*

e-mail: thomas.loerting@uibk.ac.at

Supplementary Discussion

Let's start with the calorimetric analysis of the second endotherm (ice XV→ice VI). Apparently, the second endotherm is much larger (48 J mol^{-1}) for the top two traces pertaining to H_2O ice β-XV, compared to all traces between 0.5 and 30% H_2O (7 J mol^{-1}), in which D_2O ice β-XV forms at 1.8 GPa. This prompts the question why the ice XV to ice VI transition is suppressed in D_2O ice β-XV samples. The answer is kinetics. In order to transform one ordered phase to another differently ordered phase an activation barrier needs to be overcome, where the transition state is necessarily disordered. We call this transition state ice $VI^{‡}$. This transition state then needs to order again, and turn into ice XV. Ordering of ice $VI^{‡}$ is much faster for H_2O samples than for D_2O samples. The large kinetic isotope effect is evident from the width of the first endotherm, which increases from 12±1 K in protiated samples to 18±2 K in deuterated ones (see blue double-arrows in Figure 1c). In other words, the 2.5 minutes provided for the order in ice XV to develop inside the calorimetry instrument at a scanning rate of 10 K/min are not sufficient in deuterated samples, but in protiated ones. In order to make this point more evident we have conducted a calorimetry experiment, in which the heating scan was paused for 30 minutes at 120 K. In these 30 minutes there is ample time for D_2O ice XV to form, and upon commencing the heating scan a large second endotherm of 70 J mol^{-1} is also observed for D_2O samples (see dashed green line in Figure 1a at 5% H_2O). That is, 30 minutes at 120 K are enough for deuterated ice XV to fully develop from ice β-XV, but 2.5 minutes in the temperature interval 103-128 K are not. These calorimetric findings suggest that the transition sequence is a three-step process, as follows: In the first step ice β-XV transforms to ice $VI^{‡}$ at 103 K, causing the first endotherm. In the second step, between about 103 K and 128 K ice $VI^{‡}$ develops to the more stable ordered ice XV, where kinetics are the limitation. This sequence *via* ice $VI^{‡}$ describes how the first H-order-to-H-order transition in ice physics takes place. In the third step ice XV disorders to produce metastable ice VI.

Now let's move on to the procedure for ruling out space groups using Bilbao. In order to check for the validity of a given space group, the O-atoms were reflected through each symmetry element. Within valid space groups these reflections have to match exactly with positions of other O-atoms. This point is illustrated in Supplementary Figures 1 and 2. In Supplementary Figure 1, the O-atom circled in green was analysed for space group *P*cc2. Its first reflected position can be obtained by reflection through the y-z-plane and a subsequent translation of half the lattice vector along z-direction (from -0.1 to 0.4). As we can find another O atom (circled in black) on that position, this symmetry operation is valid. Analogously, we can verify the reflection through the x-z-plane and the subsequent translation. Also, the twofold axis can be confirmed easily. In Supplementary Figure 2, we can find a different situation. Here, space group *P*nc2 is considered and the O-atom circled in green has to be reflected through the y-z-plane and subsequently translated through half of the diagonal of the a-c-face. At that position, however, we cannot find an oxygen atom. That can easily be visualized by the z-positions as the greenly circled O-atom is, again, reflected from -0.1 to 0.4, whereas the oxygen atom at the respective position within the x-y-plane is set at z= -0.4.

Let's finally analyse the statistical quality of the fits for the best four models found in our Rietveld analysis:

	wR_P (%)	χ^2
$P\bar{4}$	2.38	3.278
$Pcc2$	2.63	4.018
$P2_12_12$	2.82	4.588
$Pca2_1$	2.83	4.645

The weighted Rietveld powder R-factor, wR_P, is defined as:

$$(wR_P)^2 = \sum_i w_i[y_i(obs) - y_i(calc)]^2 \Big/ \sum_i w_i[y_i(obs)]^2 \qquad (1)$$

where the sum is over i observed points with observed and calculated intensities, y_i(obs) and y_i(calc), respectively, and a statistical weight w_i. The chi-squared (χ^2) is then defined as:

$$\chi^2 = \left(wR_P/R_{exp}\right)^2 \qquad (2)$$

The 'expected', or best-possible, or R-factor (R_{exp}) is:

$$\left(R_{exp}\right)^2 = (N - P + C) \Big/ \sum_i w_i[y_i(obs)]^2 \qquad (3)$$

Where N is the total number of observations, P is the number of refined parameters and C is the number of applied constraints.

Supplementary Figures

Supplementary Figure 1: Space group analysis for *Pcc2*. *√2 x √2 x1 super-cell of the ice VI unit cell containing the reflection conditions of Pcc2: full circles represent O-atoms, empty circles H-sites, different colours different H-sublattices and white numbers the z-positions of the O-atoms.*

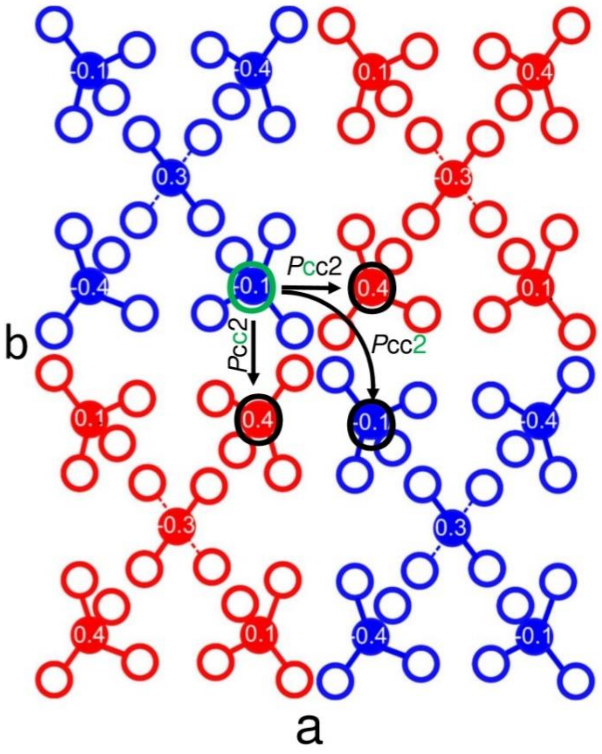

Supplementary Figure 2: Space group analysis for *Pnc2*. *√2 x √2 x1 super-cell of the ice VI unit cell containing the reflection conditions of Pnc2: full circles represent O-atoms, empty circles H-sites, different colours different H-sublattices and white numbers the z-positions of the O-atoms.*

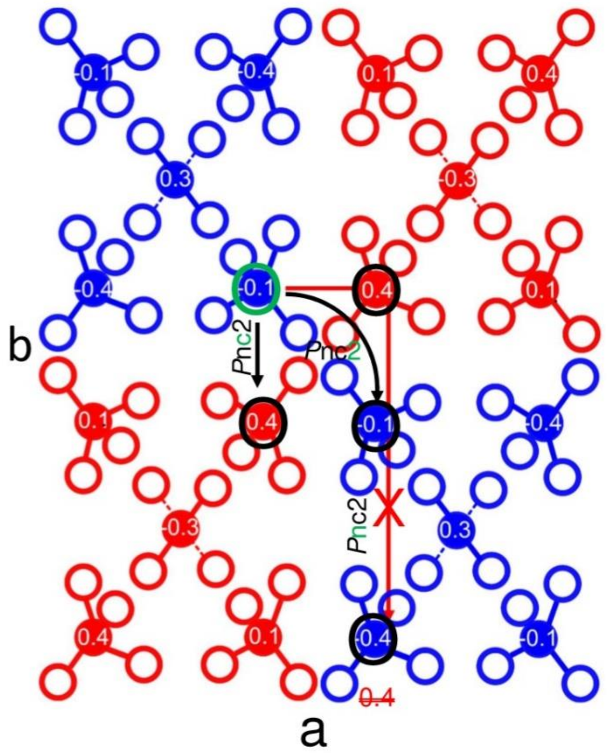

Supplementary Figure 3: Occupancies of the H-sites according to *Pcc*2 symmetry operators.

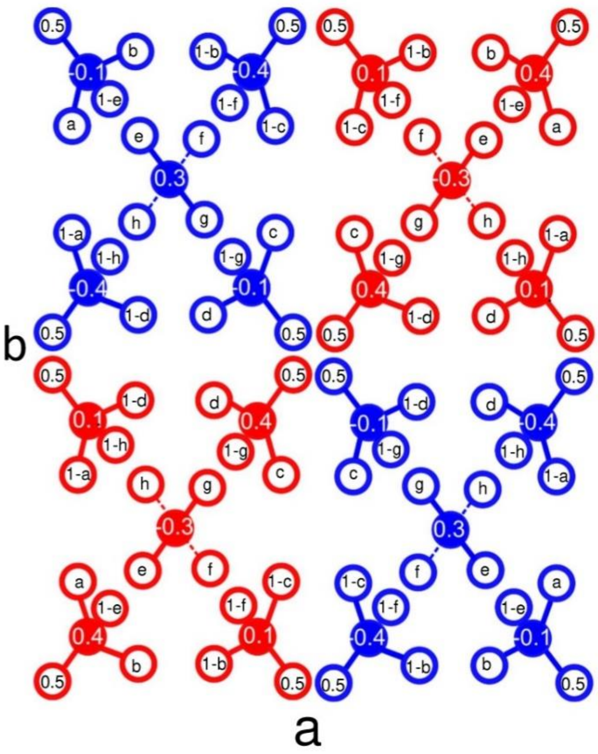

Supplementary Figure 4: Rietveld refinement of *Pcc*2 model. *Neutron powder diffraction data for ice XIX (red circular symbols) and the fit calculated from the refined Pcc2 model (green line), with the background subtracted. The difference between the model and data is represent by the purple line underneath the diffraction pattern and the positions of the Bragg peaks are indicated by vertical black tick marks. The inset shows the region between 1.7 and 2.45 Å d-spacing where peaks appear that are permitted by the ice VI cell metric but have little or no intensity in ice VI (*) and where super-lattice peaks are observed (†).*

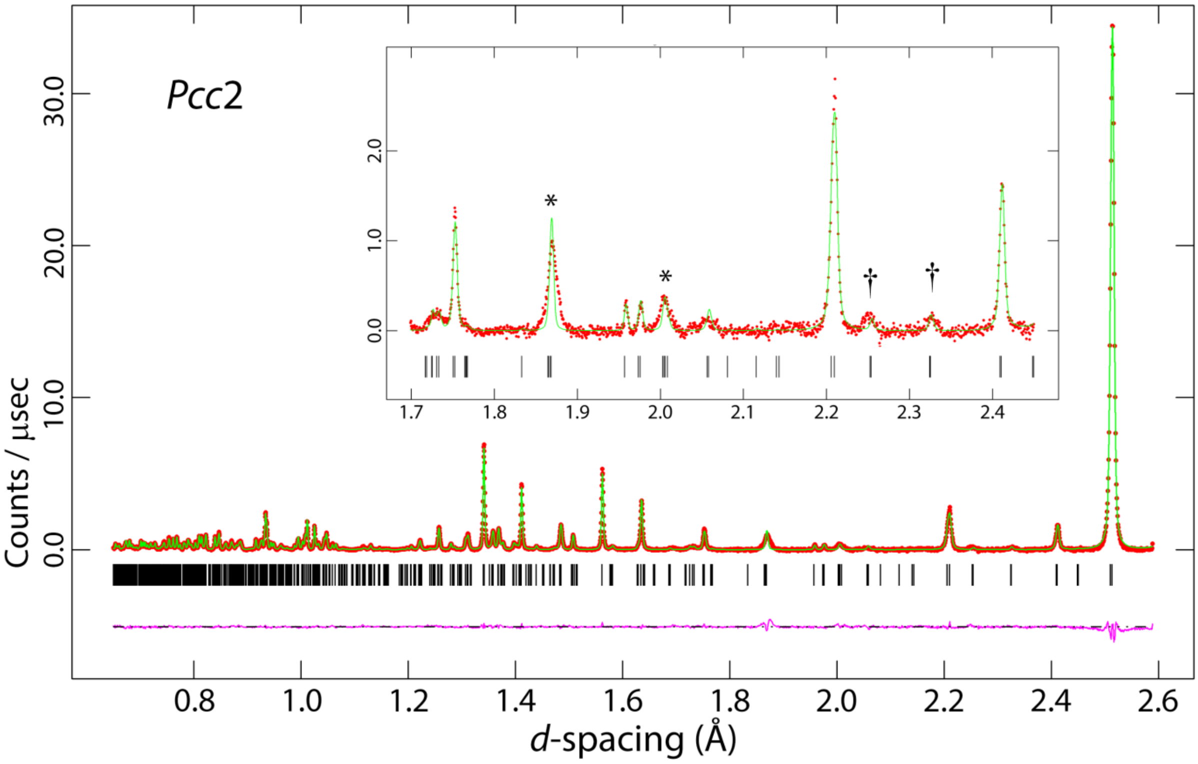

Supplementary Figure 5: Unit-cell of ice XIX (refined *Pcc*2 model). *View along the c-axis, showing the atom labelling scheme used in Supplementary Table 1.*

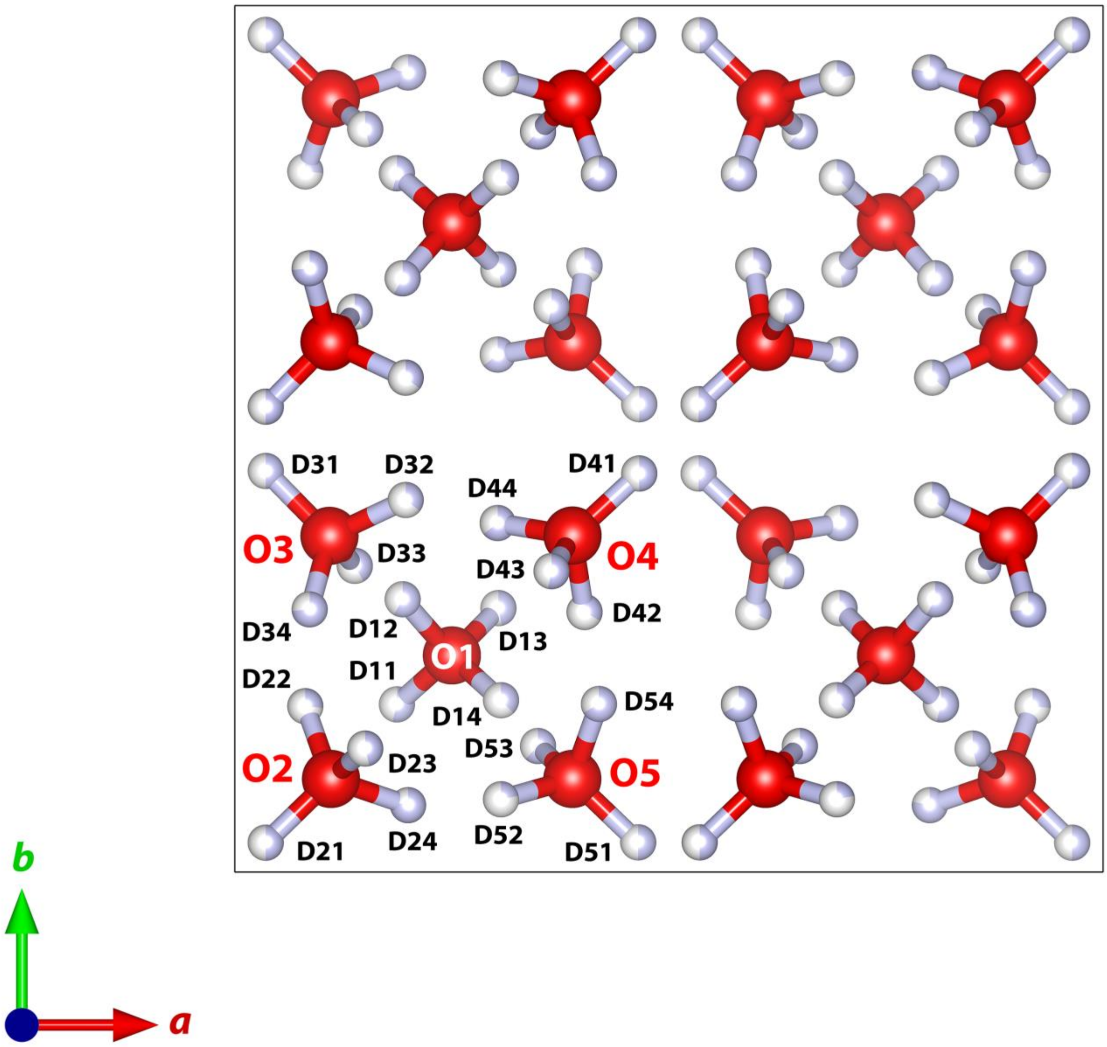

Supplementary Figure 6: Rietveld refinement of $P2_12_12$ model. *Neutron powder diffraction data for ice XIX (red circular symbols) and the fit calculated from the refined $P2_12_12$ model (green line), with the background subtracted. The difference between the model and data is represent by the purple line underneath the diffraction pattern and the positions of the Bragg peaks are indicated by vertical black tick marks. The inset shows the region between 1.7 and 2.45 Å d-spacing where peaks appear that are permitted by the ice VI cell metric but have little or no intensity in ice VI (*) and where super-lattice peaks are observed (†).*

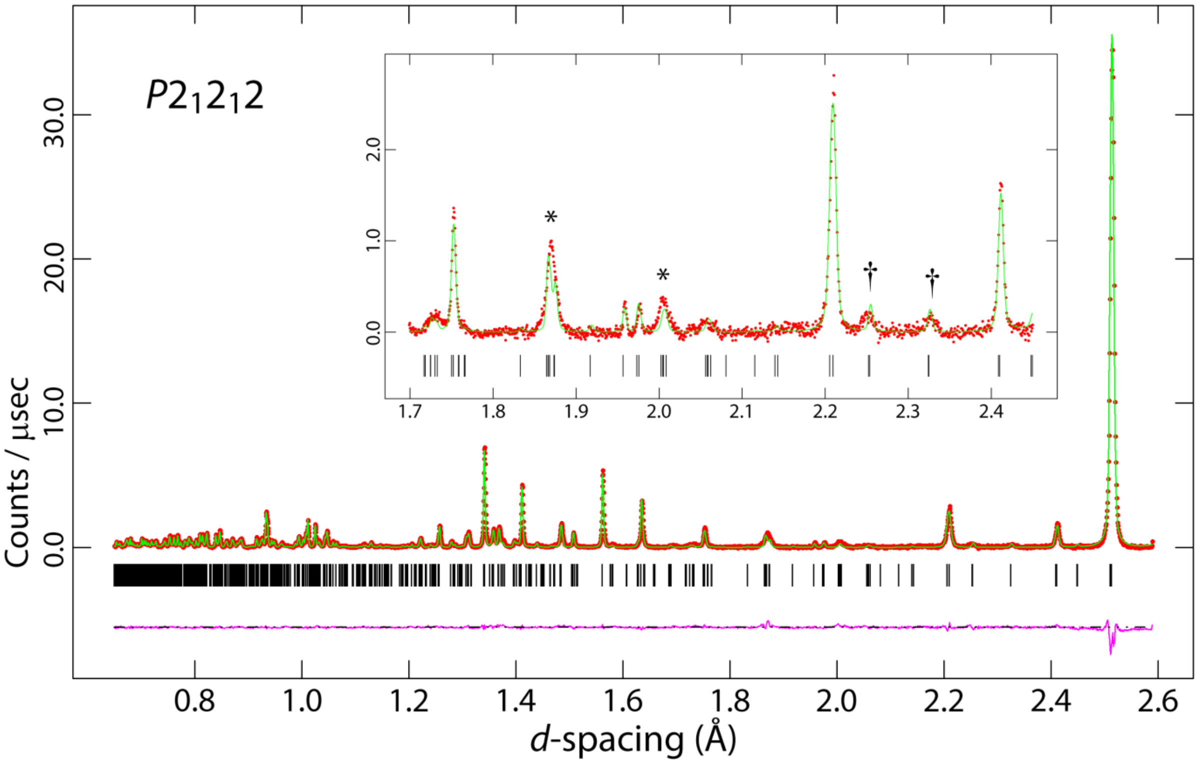

Supplementary Figure 7: Unit-cell of ice XIX (refined $P2_12_12$ model). *View along the c-axis, showing the atom labelling scheme used in Supplementary Table 2.*

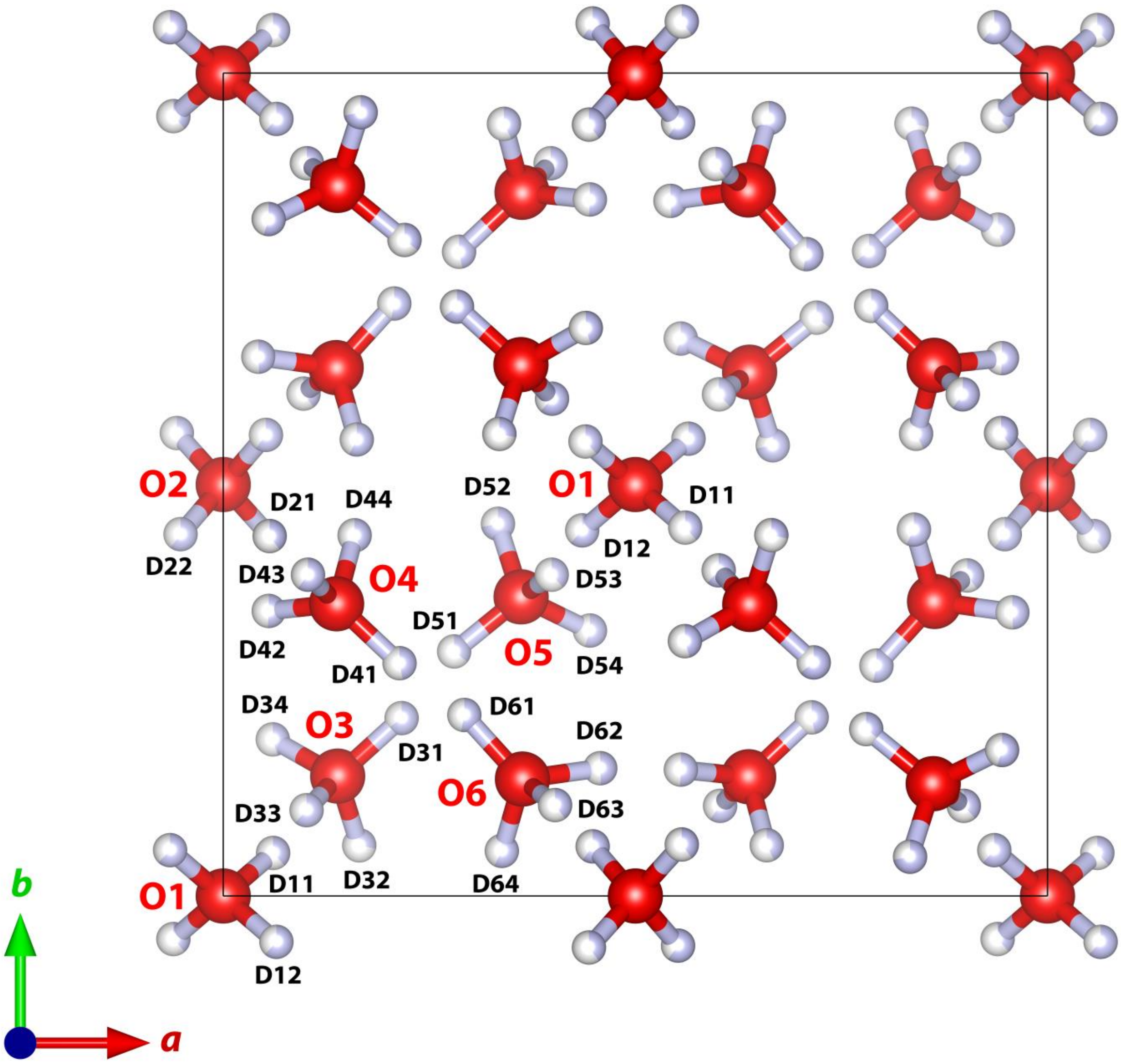

Supplementary Figure 8: Rietveld refinement of *Pca*2_1 model. *Neutron powder diffraction data for ice XIX (red circular symbols) and the fit calculated from the refined Pca2_1 model (green line), with the background subtracted. The difference between the model and data is represent by the purple line underneath the diffraction pattern and the positions of the Bragg peaks are indicated by vertical black tick marks. The inset shows the region between 1.7 and 2.45 Å d-spacing where peaks appear that are permitted by the ice VI cell metric but have little or no intensity in ice VI (*) and where super-lattice peaks are observed (†).*

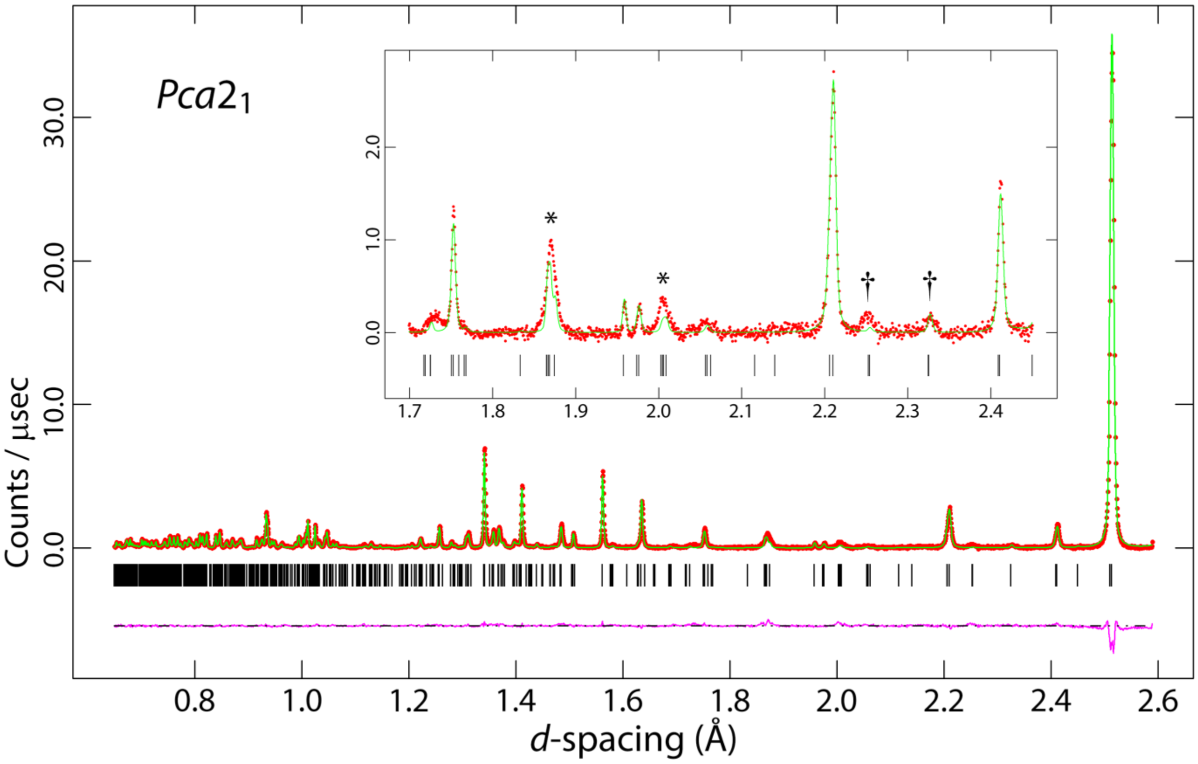

Supplementary Figure 9: Unit-cell of ice XIX (refined *Pca*2_1 model). *View along the c-axis, showing the atom labelling scheme used in Supplementary Table 3.*

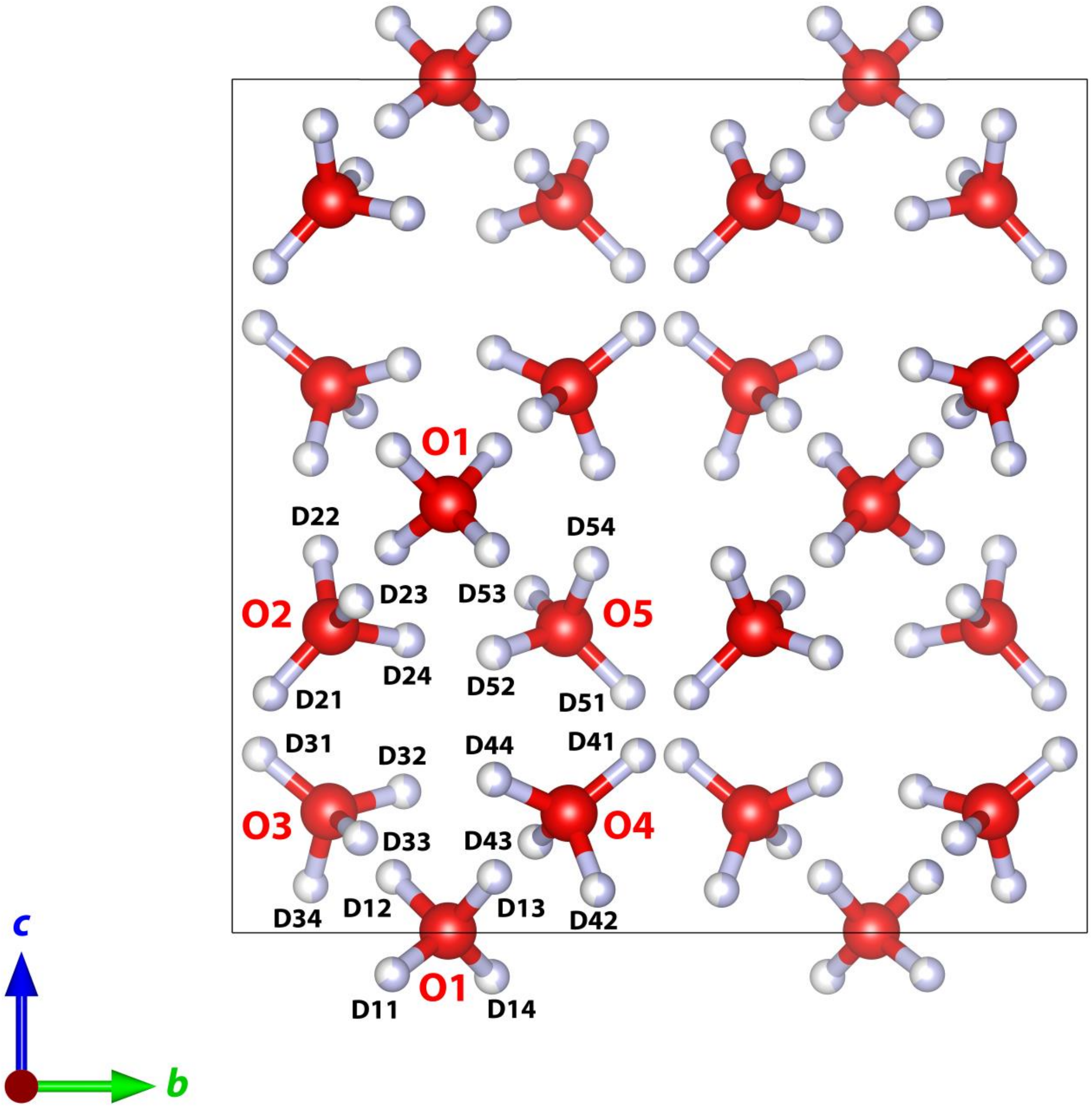

Supplementary Tables

Supplementary Table 1: Refined structure using the *Pcc*2 model

a = 8.84253 (7) Å
b = 8.82654 (7) Å
c = 5.75559 (5) Å
V = 449.218 (5) $Å^3$

Atom	Wyckoff position	*x*	*y*	*z*	Occ.	U_{iso}
O1	4*e*	0.25	0.25	0.25	1.0	0.0081
O2	4*e*	0.1107(2)	0.1074(2)	0.8770(3)	1.0	0.0081
O3	4*e*	0.1092(2)	0.3881(2)	0.6322(3)	1.0	0.0081
O4	4*e*	0.3892(2)	0.3884(2)	0.8695(3)	1.0	0.0081
O5	4*e*	0.3888(2)	0.1073(2)	0.6259(3)	1.0	0.0081
D11	4*e*	0.1869(4)	0.1933(5)	0.1555(6)	0.545(3)	0.0219
D12	4*e*	0.1931(4)	0.3143(4)	0.3424(6)	0.579(2)	0.0219
D13	4*e*	0.3036(5)	0.3046(5)	0.1400(6)	0.471(3)	0.0219
D14	4*e*	0.3066(5)	0.1981(6)	0.3595(6)	0.383(3)	0.0219
D21	4*e*	0.0356(4)	0.0340(4)	0.8910(14)	0.5	0.0219
D22	4*e*	0.0814(10)	0.1914(4)	0.7917(9)	0.271(2)	0.0219
D23	4*e*	0.1497(7)	0.1425(7)	0.0161(5)	0.455(3)	0.0219
D24	4*e*	0.1994(3)	0.0774(6)	0.8042(7)	0.783(1)	0.0219
D31	4*e*	0.0359(4)	0.4632(4)	0.6165(15)	0.5	0.0219
D32	4*e*	0.1967(4)	0.4293(7)	0.6941(10)	0.357(3)	0.0219
D33	4*e*	0.1389(8)	0.3537(8)	0.4875(5)	0.422(2)	0.0219
D34	4*e*	0.0859(7)	0.3036(3)	0.7216(6)	0.729(2)	0.0219
D41	4*e*	0.4660(4)	0.4601(4)	0.8609(13)	0.5	0.0219
D42	4*e*	0.4042(12)	0.3005(4)	0.7845(8)	0.331(3)	0.0219
D43	4*e*	0.3639(8)	0.3475(6)	0.0121(5)	0.529(3)	0.0219
D44	4*e*	0.3032(3)	0.4039(8)	0.7798(6)	0.644(3)	0.0219
D51	4*e*	0.4647(4)	0.0344(4)	0.6326(15)	0.5	0.0219
D52	4*e*	0.3065(4)	0.0847(13)	0.7192(8)	0.217(1)	0.0219
D53	4*e*	0.3496(6)	0.1451(6)	0.4887(5)	0.617(3)	0.0219
D54	4*e*	0.4189(6)	0.1939(3)	0.7048(7)	0.669(3)	0.0219

Supplementary Table 2: Refined structure using the $P2_12_12$ *model.*

a = 8.84210 (11) Å
b = 8.82682 (12) Å
c = 5.75549 (6) Å
V = 449.202 (4) $Å^3$

Atom	Wyckoff position	*x*	*y*	*z*	Occ.	U_{iso}
O1	2*a*	0.00	0.00	0.503(3)	1.0	0.0098
O2	2*b*	0.00	0.50	0.007(2)	1.0	0.0098
O3	4*c*	0.139(1)	0.146(1)	0.132(1)	1.0	0.0098
O4	4*c*	0.138(1)	0.355(1)	0.612(1)	1.0	0.0098
O5	4*c*	0.361(1)	0.364(1)	0.123(1)	1.0	0.0098
O6	4*c*	0.363(1)	0.142(1)	0.622(1)	1.0	0.0098
D11	4*c*	0.061(3)	0.052(3)	0.399(3)	0.384(1)	0.0230
D12	4*c*	0.436(2)	0.444(2)	0.404(2)	0.616(1)	0.0230
D21	4*c*	0.056(2)	0.437(2)	0.912(3)	0.400(1)	0.0230
D22	4*c*	-0.052(1)	0.440(1)	0.111(2)	0.600(1)	0.0230
D31	4*c*	0.216(1)	0.216(1)	0.143(2)	0.65(1)	0.0230
D32	4*c*	0.165(4)	0.062(2)	0.044(5)	0.28(1)	0.0230
D33	4*c*	0.101(2)	0.104(2)	0.267(2)	0.62(1)	0.0230
D34	4*c*	0.060(1)	0.190(2)	0.050(3)	0.46(2)	0.0230
D41	4*c*	0.214(2)	0.283(2)	0.613(3)	0.47(2)	0.0230
D42	4*c*	0.055(1)	0.346(2)	0.512(2)	0.51(1)	0.0230
D43	4*c*	0.103(2)	0.390(2)	0.754(2)	0.600(1)	0.0230
D44	4*c*	0.159(3)	0.438(2)	0.519(3)	0.43(1)	0.0230
D51	4*c*	0.280(1)	0.297(2)	0.132(4)	0.35(1)	0.0230
D52	4*c*	0.333(2)	0.452(1)	0.048(2)	0.72(1)	0.0230
D53	4*c*	0.399(3)	0.390(3)	0.268(2)	0.384(1)	0.0230
D54	4*c*	0.445(1)	0.323(2)	0.049(3)	0.55(2)	0.0230
D61	4*c*	0.293(1)	0.220(1)	0.614(3)	0.53(2)	0.0230
D62	4*c*	0.457(1)	0.155(2)	0.554(2)	0.49(1)	0.0230
D63	4*c*	0.403(3)	0.111(3)	0.762(2)	0.400(1)	0.0230
D64	4*c*	0.338(2)	0.055(1)	0.540(3)	0.58(1)	0.0230

Supplementary Table 3: Refined structure using the $Pca2_1$ model

a = 5.75560 (6) Å
b = 8.84224 (10) Å
c = 8.82667 (11) Å
V = 449.211 (4) $Å^3$

Atom	Wyckoff position	*x*	*y*	*z*	Occ.	U_{iso}
O1	4*a*	0.505(2)	0.253(1)	0.003(1)	1.0	0.0095
O2	4*a*	0.620(2)	0.115(1)	0.358(1)	1.0	0.0095
O3	4*a*	0.116(2)	0.113(1)	0.142(1)	1.0	0.0095
O4	4*a*	0.875(2)	0.394(1)	0.140(1)	1.0	0.0095
O5	4*a*	0.371(2)	0.389(1)	0.357(1)	1.0	0.0095
D11	4*a*	0.896(2)	0.188(1)	0.451(2)	0.65(1)	0.0223
D12	4*a*	0.422(3)	0.190(3)	0.067(2)	0.34(1)	0.0223
D13	4*a*	0.602(2)	0.307(2)	0.066(2)	0.58(2)	0.0223
D14	4*a*	0.110(3)	0.304(2)	0.449(2)	0.43(2)	0.0223
D21	4*a*	0.623(3)	0.044(2)	0.281(1)	0.58(2)	0.0223
D22	4*a*	0.530(3)	0.103(2)	0.445(2)	0.52(2)	0.0223
D23	4*a*	0.768(2)	0.146(4)	0.387(3)	0.35(1)	0.0223
D24	4*a*	0.541(3)	0.205(1)	0.343(2)	0.56(2)	0.0223
D31	4*a*	0.111(3)	0.033(2)	0.210(3)	0.42(2)	0.0223
D32	4*a*	0.034(3)	0.200(2)	0.166(3)	0.44(1)	0.0223
D33	4*a*	0.259(2)	0.149(2)	0.110(2)	0.66(1)	0.0223
D34	4*a*	0.031(4)	0.091(2)	0.055(2)	0.49(2)	0.0223
D41	4*a*	0.866(3)	0.474(2)	0.208(2)	0.48(2)	0.0223
D42	4*a*	0.941(3)	0.428(2)	0.051(1)	0.54(2)	0.0223
D43	4*a*	0.735(3)	0.355(3)	0.106(3)	0.42(2)	0.0223
D44	4*a*	0.940(3)	0.306(1)	0.179(2)	0.57(1)	0.0223
D51	4*a*	0.368(3)	0.463(2)	0.282(2)	0.52(2)	0.0223
D52	4*a*	0.462(4)	0.306(2)	0.329(2)	0.45(2)	0.0223
D53	4*a*	0.236(2)	0.350(2)	0.397(2)	0.57(2)	0.0223
D54	4*a*	0.475(3)	0.420(2)	0.431(2)	0.46(2)	0.0223
